# Intelligent diagnosis of resistance variant multiple fault locations of mine ventilation system based on ML-KNN

**DOI:** 10.1371/journal.pone.0275437

**Published:** 2022-09-30

**Authors:** Dong Wang, Jian Liu, Lijun Deng, Honglin Wang

**Affiliations:** 1 College of Safety Science & Engineering, Liaoning Technical University, Huludao, Liaoning, China; 2 Key Laboratory of Mine Thermo-Motive Disaster & Prevention, Ministry of Education, Huludao, Liaoning, China; Polytechnical Universidad de Madrid, SPAIN

## Abstract

The resistance variant faults (RVFs) observed in the mine ventilation system can utterly restrict mine safety production. Herein, a machine learning model, which is based on multi-label k-nearest neighbor (ML-KNN), is proposed to solve the problem of the rapid and accurate diagnosis of the RVFs that occur at multiple locations within the mine ventilation system. The air volume that passes through all the branches of the ventilation network, including the residual branches, was used as the diagnostic model input after the occurrence of multiple faults, whereas the label vector of the fault locations was used as the model’s output. In total, seven evaluation indicators and 1800 groups of randomly simulated faults at the typical locations in a production mine with 153 nodes and 223 branches were considered to evaluate the feasibility of the proposed model to solve for multiple fault locations diagnostic and verify the model’s generalization ability. After ten-fold cross-validation of the training sets containing 1600 groups of fault instances, the diagnostic accuracy of the model tested with the air volume of all 223 branches and the 71 residual branches’ air volume as input was 73.6% and 72.3%, respectively. On the other hand, To further evaluate the diagnostic performance of the model, 200 groups of the multiple fault instances that were not included in the training were tested. The accuracy of the fault location diagnosis was 76.5% and 73.5%, and the diagnostic time was 9.9s and 12.16s for the multiple faults instances with all 223 branches’ air volume and the 71 residual branches’ air volume as observation characteristics, respectively. The data show that the machine learning model based on ML-KNN shows good performance in the problem of resistance variant multiple fault locations diagnoses of the mine ventilation system, the multiple fault locations diagnoses can be carried out with all the branches’ air volume or the residual branches’ air volume as the input of the model, the diagnostic average accuracy is higher than 70%, and the average diagnosis time is less than one minute. Hence, the proposed model’s diagnostic accuracy and speed can meet the engineering requirements for the diagnosis of multiple fault locations for a real ventilation system in the field, and this model can effectively replace personnel to discover ventilation system failures, and also lays a good foundation for the construction of intelligent ventilation systems.

## Introduction

**Fig 1:**
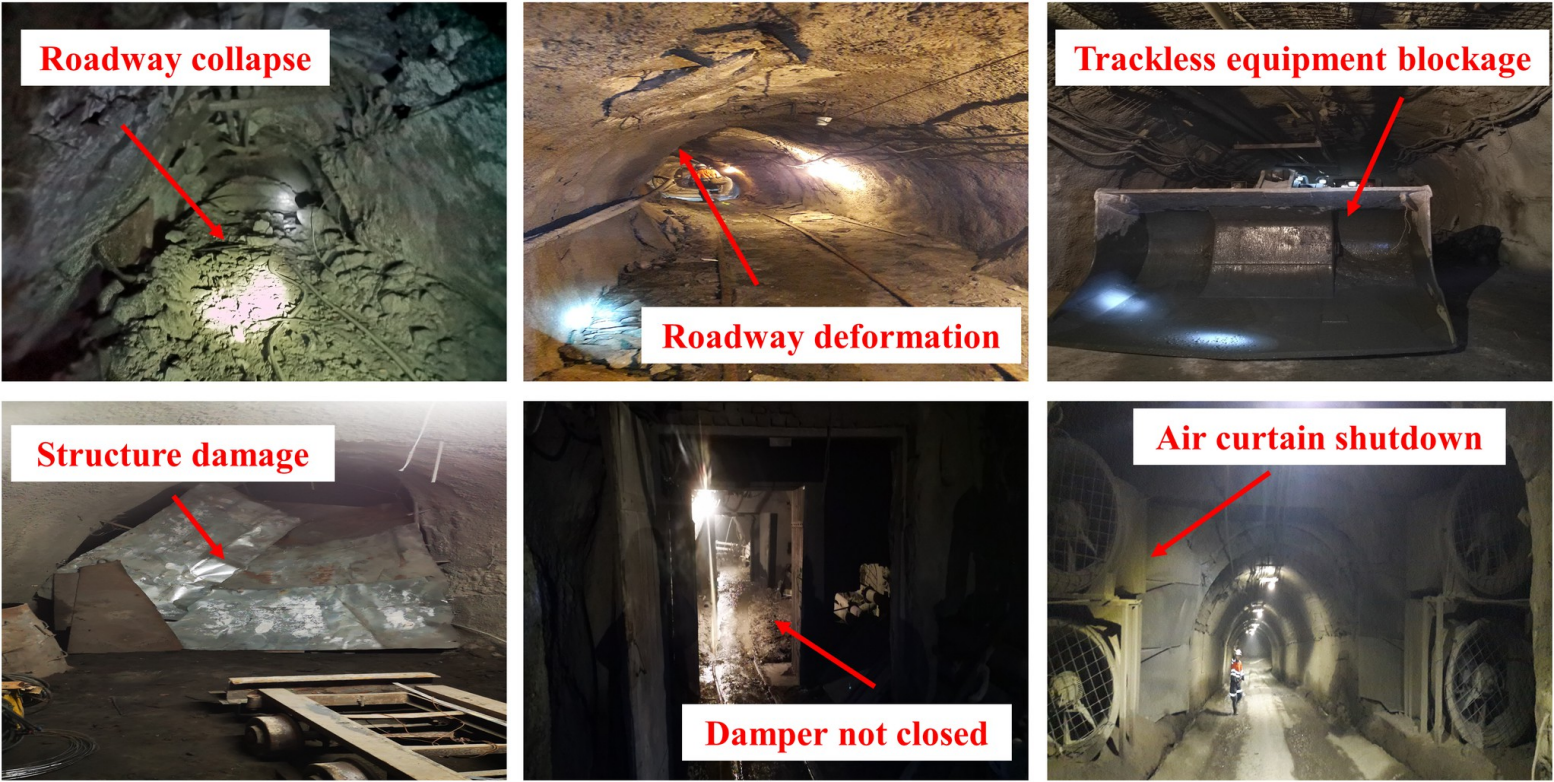
Resistance variant faults in a real mine. (Created by the author).

All abnormal deviations in the air volume of the mine ventilation system could be caused by the roadway falling deformation, dampers opening not closed or broken, fan failure, roadway extension, and scrapping, coal bunker emptying, and other factors (as shown in Fig 1) belong to the RVFs [1]. When the RVFs occur in the mine ventilation system, it extremely threatens the regular production of the mine and causes safety production issues. Currently, the majority of the mines can only rely on manual inspection to find the RVFs [2]. Furthermore, a comprehensive inspection of the mine requires at least a few days to complete, which is considered time-consuming and labor-intensive. During this time, the ventilation system remains highly risky until all the faults are investigated and dealt with [3,4]. For these reasons, an intelligent mine is needed where an intelligent ventilation system can effectively replace the human resources needed to conduct this task [5]. Intelligent diagnosis of the RVFs is one of the essential requirements for building the brain of the mine intelligent ventilation system. Its core goal is to identify the locations where the fault occurs in real-time and then deal with it according to the degree of the fault occurrence [6]. It is of great practical significance to realize the intelligent diagnosis of the RVFs to ensure the mine is safe during production and improve the ventilation system’s scientific management level.

Utilizing the theoretical concepts and best practices, the probability of failure occurring at multiple locations during the same time in a coal mine ventilation system becomes relatively small. However, the ventilation system can be overly complicated and dynamic in some mines in terms of metal mines. Moreover, the management level of the ventilation system is generally weaker than in the coal mines [7]. Therefore, it is common to notice that the RVFs of the ventilation system occur concurrently at multiple locations in metal mines [8]. Consequently, investigating the problems and identifying new rapid and accurate diagnoses of resistance variant multiple fault locations in the mine ventilation system is very essential.

The rapid development of artificial intelligence and machine learning has promoted the transformation of the mining industry from mechanization and automation to intelligence [9]. Artificial intelligence and machine learning play an important role in the construction of smart mines [10]. Much artificial intelligence and machine learning algorithms, such as deep neural networks, recurrent neural networks, and other deep learning algorithms [11,12], and heuristic algorithms such as genetic algorithms and self-encoding networks [13,14] have been applied to mine to solve practical engineering problems [15], and also played an important role in the intelligent construction of mine ventilation system. At present, these intelligent algorithms have been used to solve the key problems of mine ventilation resistance coefficient prediction and inversion [16,17], optimal adjustment of ventilation network [18,19], intelligent control of ventilation system [20], and rapid prediction of mine gas explosion and mine fire disaster parameters [21,22]. In terms of fault diagnosis, fault diagnosis technology based on artificial intelligence and machine learning has been promoted and applied in many industrial fields [23,24]. With the continuous progress and improvement of artificial intelligence technology and machine learning algorithms, it has become possible for the mine ventilation system to have the ability to intelligently diagnose faults.

Prevailing studies have shown that machine learning-related algorithms, such as supervised learning, unsupervised learning, reinforcement learning, and ensemble learning can accurately diagnose the RVFs in a mine ventilation system. Liu et al. [25–28] used the Support Vector Machine (SVM) to study the resistance variant single fault (RVSF) diagnosis of a mine ventilation system, where the air volume was the single feature and showed that the diagnostic accuracy is associated with the number of installed sensors and the degree of dispersion. The study also showed that the network topology sensitivity of the roadway, where the sensors are located, and the magnitude of the air volume in the roadway had no impact on the accurateness of the model. Meanwhile, the study showed that the diagnostic accuracy is based on the compound features of the air volume and wind pressure was higher than that which is based on the single feature of either air volume or wind pressure. Additionally, the compound features can eliminate the ill-posedness problem of the single feature. About the inability of the fault locations and fault quantities to be diagnosed synchronously, an unsupervised learning diagnosis model that does not require samples to participate in training was constructed and the Covariance Matrix Adaptation Evolution Strategy method was used to solve it. Additionally, a broom model was established to optimize the placement of the wind speed sensors for the faults diagnosis in the mine ventilation system. Moreover, the intelligent identification of the RVSF was realized using the monitoring information of the sparse wind speed sensors. Zhou et al. [29] proposed a model for the abnormal diagnosis of the ventilation system that is based on the Neural Network, which can rapidly diagnose and locate the roots and types of the abnormalities in the ventilation parameters. Zhang et al. [30] compared the accuracy of the SVM, Random Forest, and Neural Network in the diagnosis of the RVSF in the mine ventilation systems and realized that the diagnostic accuracy of the Neural Network is better than the SVM and Random Forest. Ni et al. [31] proposed an integrated model of the RVSF diagnosis of the mine ventilation system and the wind speed sensor’s optimal layout based on a Decision Tree, which solved the matching problem of the wind speed sensor’s optimal layout and diagnosis model. Huang et al. [32,33] used a Hybrid-Encoding Adaptive Evolution Strategy-based method to diagnose the RVSF of the mine ventilation system and were able to verify the advantage of this method in the calculation efficiency through comparative experiments. Additionally, a multi-objective optimal selection model of observation features was developed for the RVSF diagnosis, which has the advantage of eliminating redundant or irrelevant features. Zhou et al. [34] proposed an improved Genetic Algorithm for enhancing the penalty coefficient and kernel function parameters of the SVM, which solves the main problem of overfitting in the fault diagnosis. Gong et al. [35] established a faults diagnosis model for the mine’s local ventilation system using the Genetic Algorithm and Neural Network, which can better identify the type and location of the local ventilation system faults. Wu et al. [36] proposed a method for fault diagnosis of a local ventilation system in the coal mines that are based on the Genetic Algorithm and Rough Set Theory. Zhao et al. [37–39] established the faults roadways scope library of a ventilation system based on the Radial Basis Function Neural Network and determined the source of the locations of the faults according to the abnormal wind speed data obtained from the underground wind speed sensor. Additionally, they developed an expert system for the early warning diagnosis of the mine monitoring and control using a knowledge base and inference engine. They also established the influence matrix of the wind resistance-air flow change and obtained the branches set that affect the change in the air volume of each roadway, then judged the ventilation system faults based on the faults’ probability function of the mine roadway. Consequently, although the previous studies were able to solve the problem of the RVSF diagnosis in the mine ventilation system, the critical problem of the RVFs diagnosis that occurs in multiple locations at the same time in a mine ventilation system has not been solved yet.

The problem of the RVSF diagnosis in the mine ventilation system belongs to the single-label classification problem. In theory, the resistance variant multiple fault location diagnosis problems of the mine ventilation system can be transformed into a single fault diagnosis problem through transformation strategies. Typically, the transformation strategies comprise Binary Relevance [40], Classifier Chains [41], and Calibrated Label Ranking [42]. However, these transformation algorithms have their limitations in terms of adaptability, generalization, and computational complexity to meet the demand for rapid and accurate diagnosis of the resistance variant multiple fault locations. K-nearest neighbors (KNN) is a simple method and algorithm of machine learning [43]. The ML-KNN is a supervised machine learning multi-label classification and adaptation algorithm, which is derived from the k-nearest neighbor algorithm. This algorithm has a significant advantage that it is robust and can easily filter noisy data through the selection of the nearest neighbors *K*. As compared with the multi-label classification algorithms, such as Boostexter [44], Adtboost. MH [45], and Rank-SVM [46], this algorithm has significant improvements in terms of accuracy, performance, and efficiency [47], and has been successfully applied in the field of mechanical compound faults diagnosis [48], medicine [49], text categorization [50], which is suitable for the case of the multiple fault locations diagnosis of a mine ventilation system. In this study, a supervised learning sample of the mine ventilation system resistance variant for multiple faults was obtained by simulation, and a location diagnosis machine learning model of the resistance variant multiple faults of the mine ventilation system was constructed. The ML-KNN algorithm was chosen to solve the model, and its feasibility and reliability were confirmed by a production mine simulation test.

## Multiple fault locations diagnosis model and solution

### Problem formulation

The essence of the RVFs in the mine ventilation system is a sudden change of the wind resistance in the faulty branches, which is intuitively manifested as the abnormal change in the air volume of the ventilation system. The fault location diagnosis of the mine ventilation system determines the locations of the faults according to the performance information of the ventilation system, such as air volume that can be monitored. This study only considers the situation where the RVFs occur in two or more locations in the ventilation system at the same time.

In the mine ventilation network, which can be described as [51]

$$\left\{ \begin{array}{l} G=(V,E)\\ V=\{v_{1},v_{2},\cdots ,v_{m}\}\\ E=\{e_{1},e_{2},\cdots ,e_{n}\} \end{array}\right.$$ (1)

where ***v*** is the set of *m* nodes and ***E*** is the set of *n* branches.

When multiple faults occur in the ventilation system, the topological relationship of the ventilation network ***G*** does not change, unlike the wind resistance of the faulty branches which changes significantly. Therefore, the ventilation system follows the common basic laws whether there are multiple faults or only a single or no faults. Hence, it satisfies the flux equilibrium equation of the node, resistance equilibrium of the circuit, and the law of resistance [52]. Let $Q^{\prime }=\{q_{1}^{\prime },q_{2}^{\prime },\cdots ,q_{n}^{\prime }\}$ be the vector that corresponds to the air volume set of the branches after the occurrence of multiple faults. Let $H^{\prime }=\{h_{1}^{\prime },h_{2}^{\prime },\cdots ,h_{n}^{\prime }\}$ be the vector that corresponds to the wind pressure set of the branches after the occurrence of multiple faults. Hence, the ventilation system satisfies the following constraints:

$$\left\{ \begin{array}{l} B{Q^{\prime }}^{T}={\left(\sum_{j=1}^{n}b_{ij}q_{j}^{\prime }\right)}_{m\times 1}=0\\ C{H^{\prime }}^{T}-{H^{\prime \prime }}^{T}={\left(\sum_{j=1}^{n}C_{ij}h_{j}-h_{i}^{\prime \prime }\right)}_{s\times 1}=0 \end{array}\right.$$ (2)

where $B={\left(b_{ij}\right)}_{m\times n}$ is the ventilation network complete incidence matrix, $C={\left(c_{ij}\right)}_{s\times 1}$ is the ventilation network circuit matrix, *s* is the number of circuits, and *H*″ is the additional resistance of the circuits.

The mine ventilation system resistance variant for multiple fault location diagnoses can be regarded as a multi-label classification problem. Assuming that the ventilation system occurs *p* times the resistance variant for multiple faults, and considering that after the occurrence of each fault, the set of branches for air volume or pressure constitutes a fault sample instance *x*, so the fault instance domain ***X*** = {*x*_1_,*x*_2_,⋯,*x*_*p*_} will be formed. Let ***L*** = {*L*_1_,*L*_2_,⋯,*L*_*r*_} be the label space that is composed of all the multiple fault categories and *r* is the total number of the labels used. The resistance variant for multiple fault locations diagnosis training set $T=\{(x_{i},L_{i})|1\leq i\leq p,x_{i}\in X,L_{i}\subseteq L\}$ consists of the fault sample instances and their corresponding category labels.

**Fig 2:**
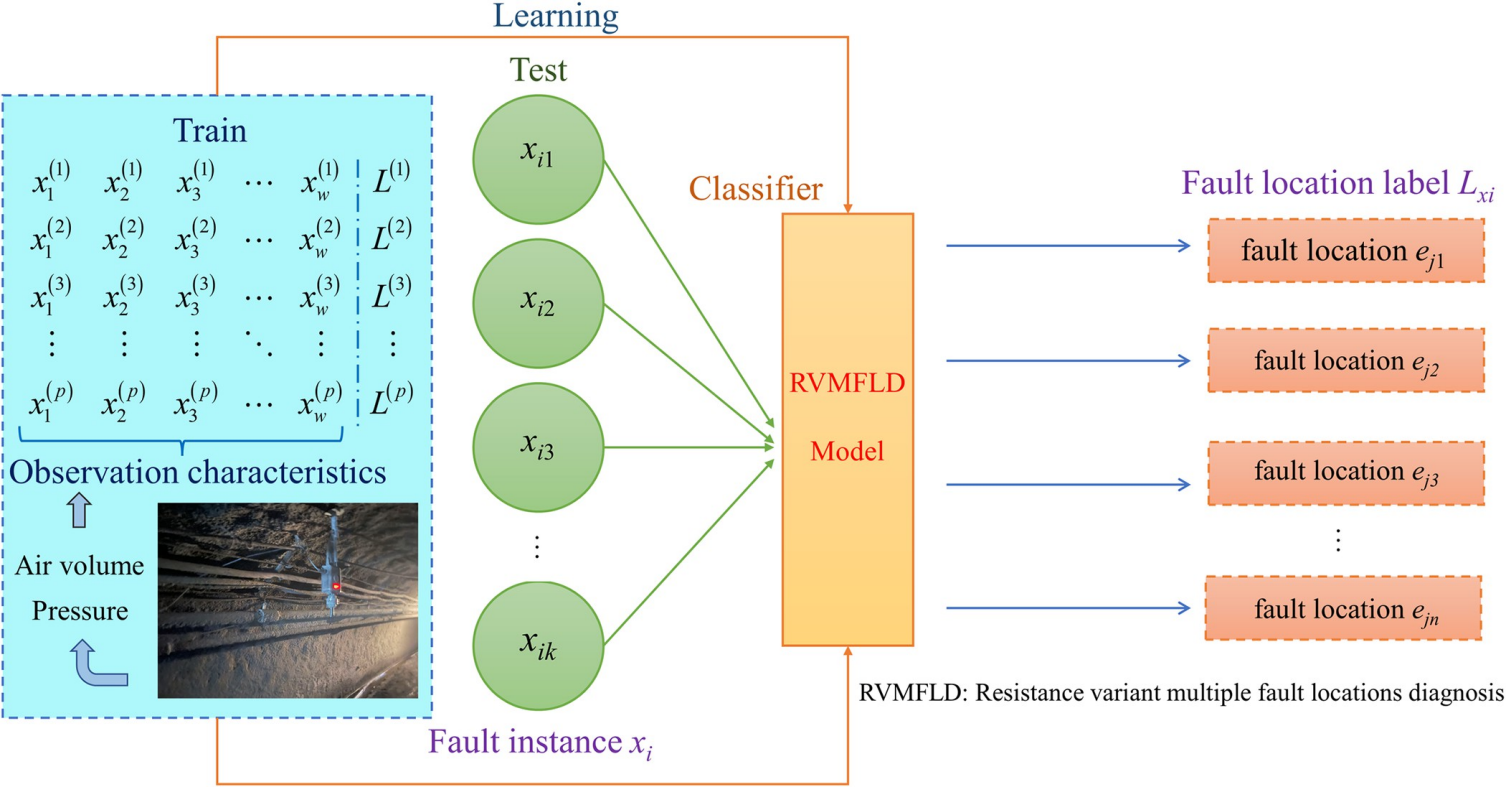
The machine learning model of the resistance variant for multiple fault location diagnoses.

The main aim of the multiple fault location diagnosis problems is to acquire a real-valued classification function *f*:***X***×***L***→***R*** through machine learning of the training set ***T***. To determine the category label *L*_*x*_ that is contained in the multiple faults attribute sample x according to this classification, the locations of the multiple faults should be determined to construct a multiple fault locations diagnostic classifier. The machine learning model for the resistance variant for multiple fault locations diagnostic is shown in Fig 2. Fig 2 describes the process of fault sample generation, model training, and fault diagnosis. The generation of the fault sample set is acquired by actual testing or simulation, and the process of model training and fault diagnosis is completed by the ML-KNN machine learning algorithm.

Let ${\vec{L}}_{x}$ be the category vector of *x*, where its element ${\vec{L}}_{x}(l)(l\in L)$ is equivalent to the value of 1 (if *l*∈*L*_*x*_) or 0. *N*(*x*) denotes the *K* nearest neighbors of *x* that is obtained in the training set. Based on the nearest neighbor label sets, a membership counting vector, that calculates the number of nearest neighbors of the instance *x* belonging to the *l*^th^ class, is introduced and can be defined as

$${\vec{C}}_{x}(l)=\sum_{a\in N(x)}{\vec{L}}_{a}(l),\;l\in L$$ (3)

For any new fault instance *t*, let $H_{1}^{l}$ be the case where *t* has a label *l* and $H_{0}^{l}$ be the case where *t* does not have a label *l*. $E_{j}^{l}(j\in 0,1,\cdots ,K)$ denotes the case where there are *j* events that contain *l* label, among the *K* nearest neighbors of *t*. Therefore, and according to the maximum a posteriori probability and the Bayesian rule [53], the objective function of the resistance variant for multiple fault locations diagnoses can be obtained and is defined as follows:

$${\vec{L}}_{t}(l)=\underset{b\in \{0,1\}}{\arg\;\max}\;P(H_{b}^{l})P(E_{j}^{l}|H_{b}^{l})$$ (4)

where $P(H_{b}^{l})$ is the prior probability of *t* containing the *l* label and $P(E_{j}^{l}|H_{b}^{l})$ is the posterior probabilities that are calculated as follows:

$$\left\{ \begin{array}{l} P(H_{0}^{l})=1-P(H_{1}^{l})\\ P(H_{1}^{l})=\frac{s+\sum_{i=1}^{p}{\vec{L}}_{x_{i}}(l)}{s\times 2+p} \end{array}\right.$$ (5)

where *s* is a smoothing coefficient that is equivalent to 1,

$$\left\{ \begin{array}{l} P(E_{j}^{l}|H_{0}^{l})=\frac{s+c^{\prime }[j]}{s\times (K+1)+\sum_{g=0}^{K}c[g]}\\ P(E_{j}^{l}|H_{1}^{l})=\frac{s+c[j]}{s\times (K+1)+\sum_{g=0}^{K}c[g]} \end{array}\right.$$ (6)

where *c*[*j*] and *c*′[*j*] calculates the number of training instances with and without *l* label, respectively, whose K-nearest neighbors contain *j* instances with label *l* exactly.

### Multiple fault location diagnosis solution algorithm

**Fig 3:**
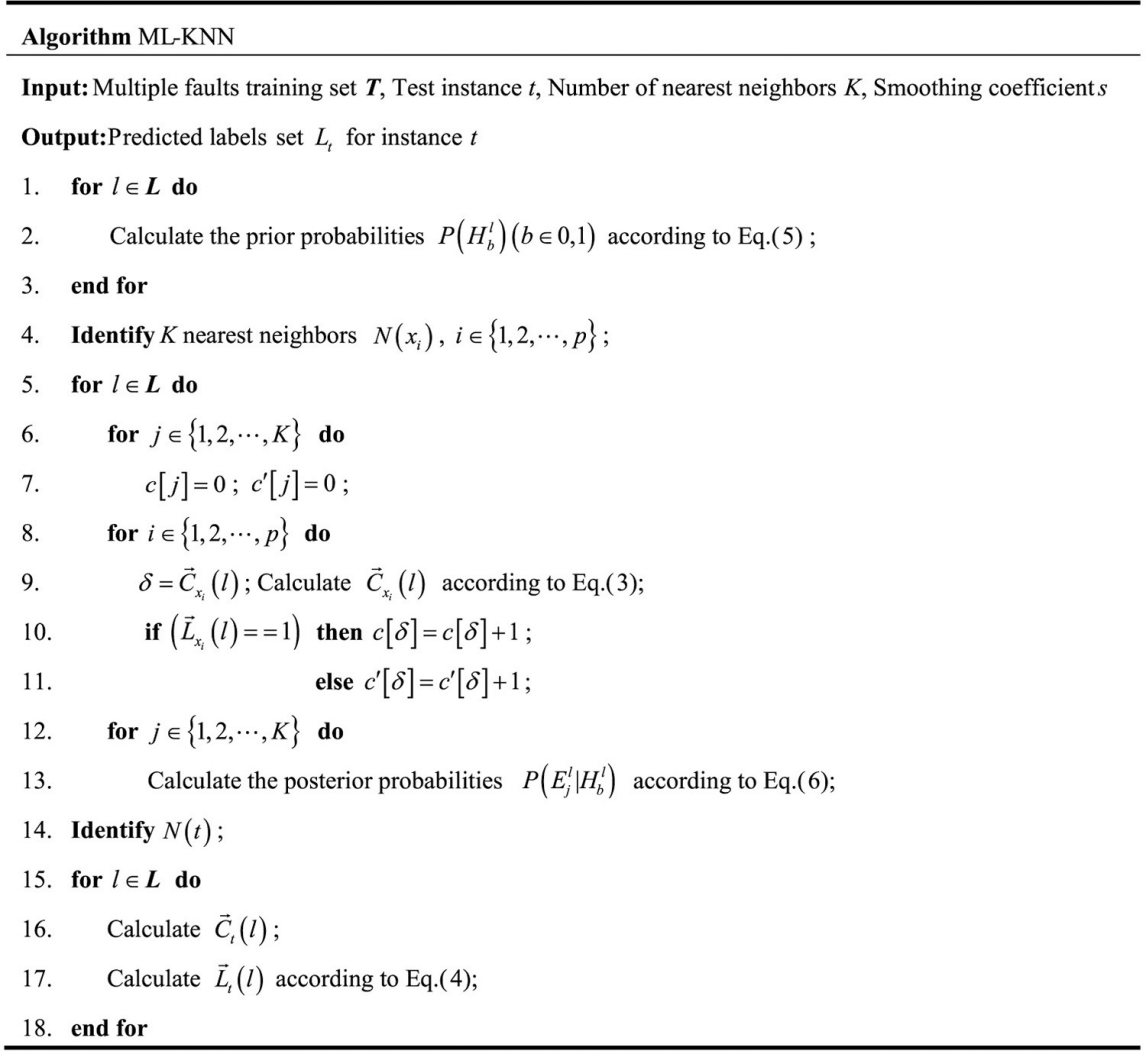
Pseudo-code of the ML-KNN model.

The ML-KNN algorithm was used to solve the objective function of the mine ventilation resistance variant for multiple fault location diagnoses. For the unknown fault tag instances, the ML-KNN algorithm first identifies the *K* nearest neighbors in the training set. The label set of these adjacent instances is determined according to the maximum posterior principle that is based on the number of adjacent instances in each possible class, which are obtained from the label set of these adjacent instances [47]. In this paper, the distance between the instances is measured by the Euclidean metric. The ML-KNN algorithm is described in Fig 3.

## Experiments

### Production mine ventilation system

**Fig 4:**
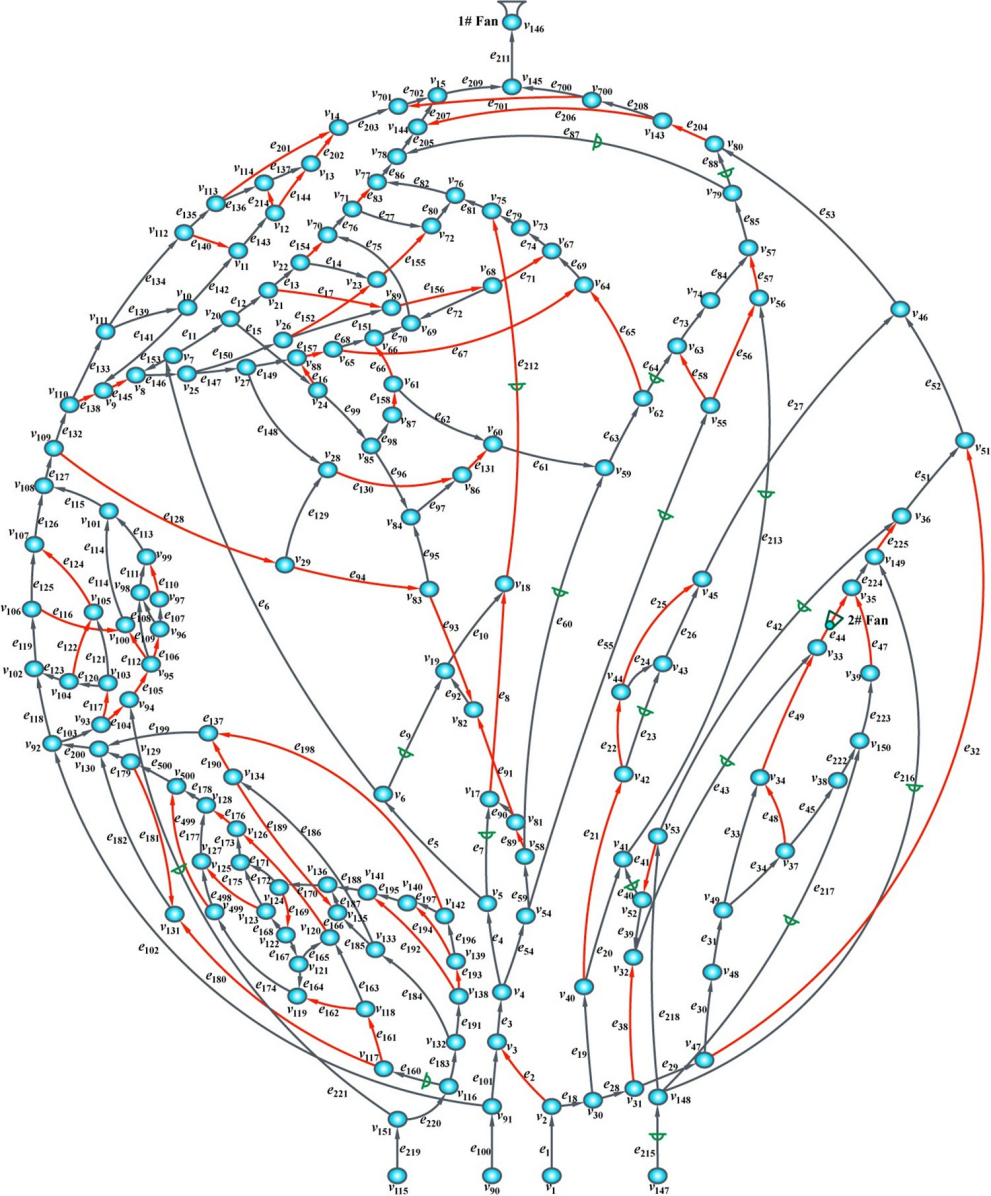
Metal production mine ventilation network. (Created by the author).

A metal production mine was used as an example for multiple faults diagnosis experiments to verify the effectiveness and reliability of the ML-KNN algorithm in the resistance variant for multiple fault locations diagnoses of the mine ventilation system. The ventilation network of the production mine is shown in Fig 4, with 153 nodes and 223 branches. The 1# fan is the main ventilation fan of the mine and is installed in branch *e*211, whereas the 2# fan is the underground ventilation fan station and is installed in branch *e*44. The characteristic curve equations of the two fans are *h*_1_(*q*) = 2787.7+17.522*q*-0.0887*q*^2^ and *h*_2_(*q*) = 444.73+26.2919*q*-2.1719*q*^2^. Overall, there are 18 dampers in the mine. The residual branches are marked with a red symbol, the dampers are marked with a green symbol, and the nodes are marked with a blue symbol.

The situation where the dampers are opened and not closed or broken leads to a decrease in the equivalent wind resistance of the roadway due to the reduced resistance type fault. On the other hand, the situation where the roadway is deformed or blocked by some trackless equipment leads to an increase in the roadway wind resistance due to the escalated resistance type fault. These two fault types are frequently observed in this metal mine. Therefore, some roadways with dampers and main roadways were selected for the experiments. The branches *e*_9_, *e*_55_, *e*_60_, *e*_64_, *e*_87_, *e*_88_, *e*_212_, *e*_213_, and *e*_221_ were selected to create multiple faults of descending resistance by opening the two dampers at different degrees. The other branches *e*_6_, *e*_21_, *e*_53_, *e*_65_, *e*_102_, *e*_128_, and *e*_203_ were selected to create multiple faults of increasing resistance by adding the wind resistance at different degrees. In the two roadways, multiple faults are constructed of increasing and decreasing resistance simultaneously. The details of these fault test branches are shown in Table 1.

**Table 1:** Parameters of the fault test branches.

Serial numbers of branches	Start-end nodes	*R*/(*N*∙*s*^2^∙*m*^-8^)	*Q*/m^3^∙s^-1^	With dampers	Fault type
*e*_9_	(*v*_6_,*v*_19_)	3014.887	0.10	Yes	Reducing resistance
*e*_55_	(v_54_,v_55_)	14.736	5.39	Yes	Reducing resistance
*e*_60_	(*v*_58_,*v*_59_)	10.067	6.18	Yes	Reducing resistance
*e*_64_	(*v*_62_,*v*_63_)	4.514	5.16	Yes	Reducing resistance
*e*_87_	(*v*_79_,*v*_78_)	0.290	28.64	Yes	Reducing resistance
*e*_88_	(*v*_90_,*v*_80_)	1.283	-6.37	Yes	Reducing resistance
*e*_212_	(*v*_18_,*v*_75_)	1.186	23.93	Yes	Reducing resistance
*e*_213_	(*v*_41_,*v*_56_)	5.259	11.73	Yes	Reducing resistance
*e*_221_	(*v*_151_,*v*_94_)	1.081	7.49	Yes	Reducing resistance
*e*_6_	(*v*_6_,*v*_7_)	0.006	46.52	No	Increasing resistance
*e*_21_	(*v*_40_,*v*_42_)	0.824	19.67	No	Increasing resistance
*e*_53_	(*v*_46_,*v*_80_)	0.032	83.13	No	Increasing resistance
*e*_65_	(*v*_62_,*v*_64_)	1.020	18.38	No	Increasing resistance
*e*_102_	(*v*_91_,*v*_92_)	0.009	31.79	No	Increasing resistance
*e*_128_	(*v*_109_,*v*_29_)	0.496	7.51	No	Increasing resistance
*e*_203_	(*v*_14_,*v*_701_)	0.076	34.30	No	Increasing resistance

The change of the ventilation system air volume after the occurrence of the multiple faults was simulated by the Mine Ventilation Simulation System (MVSS) [54] using the method of randomly created resistance variables. A total of 1800 groups of multiple fault instances were generated, of which 1600 groups were randomly selected as the multiple faults sample training sets and the remaining 200 sets of untrained fault instances were used as the multiple faults diagnosis test sets. Experimentally, the air volume of all the branches in the ventilation network and the air volume of the 71 remaining branches were used as the available data, and the label vector, that is composed of the faulty branches was used as the output to generate a ventilation system resistive-variable for multiple fault locations diagnostic classifier. The inputs to the classifier are normalized using the Min-Max normalization method, as shown in Eq (7).


$$x^{*}=\frac{x-\min}{\max-\min}$$ (7)

The fault sample set for the resistance variant multiple fault locations diagnoses of the mine ventilation system is shown in Table 2.

**Table 2:** Fault sample set.

Sample number	$q_{1}^{\prime }$	$q_{2}^{\prime }$	$q_{3}^{\prime }$	$q_{4}^{\prime }$	$q_{5}^{\prime }$	…	$q_{700}^{\prime }$	$q_{701}^{\prime }$	$q_{702}^{\prime }$	*e*_*i*_, *e*_*j*_
1	99.17	16.85	66.48	14.26	14.05	…	61.75	-0.87	33.14	*e*_6_, *e*_21_
2	97.23	16.75	61.97	5.46	5.22	…	61.37	-1.16	32.94	*e*_6_, *e*_21_
3	96.29	16.79	60.73	2.81	2.56	…	61.20	-1.30	32.86	*e*_6_, *e*_21_
…	…	…	…	…	…	…	…	…	…	…
1798	103.61	15.83	80.76	42.14	42.03	…	55.05	15.63	21.56	*e*_221_, *e*_203_
1799	102.92	16.40	81.32	43.05	42.95	…	56.35	12.46	24.84	*e*_221_, *e*_203_
1800	101.39	17.63	82.86	45.26	45.17	…	59.81	4.43	30.75	*e*_221_, *e*_203_

### Algorithm and performance evaluation indicator

In terms of the selection of evaluation indicators for multi-label classification models, previous researchers Wu et al. [55], Zhu et al. [56], and Wang et al. [57] used hamming loss, ranking loss, coverage, average precision, and one-error to evaluate the model. On this basis, we increase the accuracy and F1 score [58] to help us evaluate the predictive performance of the model. Multiple fault locations diagnosis algorithm and performance are judged by the seven evaluation indicators, which are defined as follows [59]:

(1) Accuracy: calculates the proportion of the correctly classified samples to the total number of samples. The higher the accuracy, the better the prediction performance. Let ${\hat{y}}_{i}$ be the predicted label of the *i*^th^ sample, *y*_*i*_ be the true label of the sample, and *N* be the number of the predicted samples. The accuracy can be calculated as follows:

$$Acc(y,\hat{y})=\frac{1}{N}\sum_{i=0}^{N-1}1({\hat{y}}_{i}=y_{i})$$ (8)

where 1(*x*_*i*_) is the indicator function.

(2) Hamming loss: calculates the proportion of the incorrectly predicted labels. The smaller the hamming loss, the better the prediction performance. This can be calculated as follows:

$$HL(y,\hat{y})=\frac{1}{M}\sum_{i=0}^{M-1}1({\hat{y}}_{i}\neq y_{i})$$ (9)

where *M* is the number of labels.

(3) Ranking loss: examines the cases of misordering in the sorted sequence of category labels in the sample, and this loss averages the number of the misordered label pairs in the sample. The smaller the ranking loss, the better the prediction performance. *y*∈{0,1}^*N*×*M*^ denotes the binary label matrix of the real labels and $\hat{f}\in R^{N\times M}$ denotes the score associated with each of its labels. It can be calculated as follows:

$$RL(y,\hat{f})=\frac{1}{N}\sum_{i=0}^{N-1}\frac{1}{{\left\|y_{i}\right\|}_{0}(M-{\left\|y_{i}\right\|}_{0})}|\{\left(k,l):{\hat{f}}_{ik}\leq {\hat{f}}_{il},y_{ik}=1,y_{il}=0\right\}|$$ (10)

where |⋅| calculates the number of elements in the set and ‖⋅‖_0_ calculates the number of non-zero elements in the vector.

(4) Coverage: examines the search depth required to cover all the relevant tokens in the sorted sequence of the category tokens of a sample. The lower the coverage, the better the prediction. It can be calculated as follows:

$$Cov(y,\hat{f})=\frac{1}{N}\sum_{i=0}^{N-1}\underset{j:yij=1}{\max}\;rank_{ij}-1$$ (11)

where ${\mathrm{rank}}_{ij}=|\left\{k:{\hat{f}}_{ik}\geq {\hat{f}}_{ij}\right\}|$.

(5) Average precision: examines the case where the tag that comes before the relevant tag in the sorted sequence of the category tags of the sample remains the relevant tag. The higher the average precision, the better the prediction performance. It can be calculated as follows:

$$AP(y,\hat{f})=\frac{1}{N}\sum_{i=0}^{N-1}\frac{1}{{\left\|y_{i}\right\|}_{0}}\sum_{j:yij=1}\frac{\left|L_{ij}\right|}{{\mathrm{rank}}_{ij}}$$ (12)

where $L_{ij}=|\left\{k:y_{ik}=1,{\hat{f}}_{ik}\geq {\hat{f}}_{ij}\right\}|$.

(6) One-error: examines the case where the labels at the front end of the sequence are not part of the set of the related labels in the sorted sequence of the category labels of the sample. The smaller the One-error, the better the prediction performance. It can be calculated as follows:

$$OE(y,\hat{f})=\frac{1}{N}\sum_{i=0}^{N-1}1\{[\arg\;\max\hat{f}]\notin y_{i}\}$$ (13)

(7) F1 score: a harmonic mean of the precision and recall, where an F1 score reaches its best value at 1 and worst score at 0. It can be calculated as follows:

$$F1(y,\hat{y})=\frac{1}{N}\sum_{i=0}^{N-1}\frac{\left|{\hat{y}}_{i}\cap y_{i}\right|}{\left|{\hat{y}}_{i}|+|y_{i}\right|}$$ (14)

## Results and discussion

To evaluate the performance of the ML-KNN algorithm and model in multiple fault locations diagnosis of the mine ventilation system, ten-fold cross-validation was performed on the diagnostic training set, and the mean ± the standard deviation for each result was selected as the result of the evaluation indicator, as shown in Tables 3 and 4.

**Table 3:** Cross-validation results of the resistance variant for multiple fault location diagnoses.

	*K* = 1	*K* = 2	*K* = 3	*K* = 4	*K* = 5
Accuracy	**0.763±0.032**	0.734±0.030	0.751±0.029	0.728±0.029	0.704±0.026
Hamming loss	0.030±0.004	0.025±0.004	0.025±0.003	0.024±0.003	**0.024±0.002**
Ranking loss	0.061±0.013	**0.056±0.012**	0.057±0.010	0.057±0.008	0.058±0.009
Coverage	2.502±0.331	**2.466±0.311**	2.484±0.289	2.483±0.236	2.527±0.241
Average precision	0.815±0.049	**0.917±0.017**	0.911±0.014	0.905±0.012	0.896±0.014
One-error	0.111±0.041	**0.012±0.007**	0.017±0.007	0.021±0.009	0.029±0.016
F1 score	0.883±0.021	0.889±0.018	0.894±0.018	**0.895±0.015**	0.894±0.011

**Table 4:** Cross-validation results of the resistance variant for multiple fault location diagnoses.

	*K* = 1	*K* = 2	*K* = 3	*K* = 4	*K* = 5
Accuracy	**0.747 ± 0.027**	0.726 ± 0.029	0.731 ± 0.029	0.710 ± 0.040	0.701 ± 0.036
Hamming loss	0.032 ± 0.004	0.028 ± 0.004	0.026 ± 0.004	**0.023 ± 0.004**	0.025 ± 0.003
Ranking loss	0.072 ± 0.011	0.062 ± 0.009	0.060 ± 0.009	**0.059 ± 0.009**	0.062 ± 0.010
Coverage	2.818 ± 0.278	2.615 ± 0.225	2.569 ± 0.250	**2.543 ± 0.234**	2.626 ± 0.238
Average precision	0.810 ± 0.050	**0.909 ± 0.013**	0.903 ± 0.014	0.900 ± 0.012	0.887 ± 0.014
One-error	0.115 ± 0.050	**0.013 ± 0.006**	0.017 ± 0.005	0.019 ± 0.007	0.026 ± 0.011
F1 score	0.879±0.021	0.879±0.017	0.889±0.018	**0.893±0.019**	0.890±0.017

Table 3 shows the cross-validation results of multiple fault locations diagnosis when all the branches’ air volumes of the ventilation network were used as the model inputs. The value of the number of the nearest neighbor *K* of the sample will have a greater impact on the results of the algorithm [43]. A small value of *K* means that only the training instances that are close to the input instance will affect the prediction result, but it is prone to overfitting. If the value of *K* is too large, the advantage is that the estimation error of learning can be reduced, but the disadvantage is that the approximate error of learning increases. At this time, the training instance far from the input instance will also affect the prediction, making the prediction wrong [60,61]. Therefore, we choose the value range of *K* to be 1–5 for research. The effects of different *K* values on the cross-validation results were also compared. When *K* = 1, the model predicted the highest accuracy. When *K* = 2, the model predicted the lowest ranking loss, coverage, and one-error, while recording the highest average precision. When *K* = 4, the predicted F1 score was the highest. When *K* = 5, the predicted hamming loss was the lowest. The average prediction accuracy of the model is 73.6%, the average hamming loss is 2.56%, the average ranking loss is 5.78%, the average coverage is 249.24%, the average precision is 88.88%, the average one-error is 3.80%, and the average F1 score is 0.891.

The accuracy of the diagnostic results is given more consideration when diagnosing multiple fault points in the mine ventilation system. When *K* = 1, the diagnostic accuracy was the best. However, the model was also the most complicated, prone to overfitting, and easy to learn the noise to ignore the real distribution of the data. In a comprehensive comparison, *K* = 2 showed a better prediction performance. The diagnosis model of *K* = 2 was used to diagnose 200 sets of fault instances that were not involved in the training. The diagnosis found that among the 200 sets of the fault instances, 153 groups of the fault locations were correctly diagnosed, with a diagnostic accuracy rate of 76.5% and a diagnosis time of 9.90s.

Table 4 shows the cross-validation results of the multiple fault locations diagnosis when the air volume of 71 remaining branches is used as the model inputs. When *K* = 1, the model predicted the highest accuracy. *W*hen *K* = 2, the model prediction had the highest average precision and the lowest one-error. When *K* = 4, the model predicted the lowest hamming loss, ranking loss, and coverage, while recording the highest F1 score. The average prediction accuracy of the model is 72.3%, the average hamming loss is 2.68%, the average ranking loss is 6.30%, the average coverage is 263.42%, the average precision is 88.18%, the average one-error is 3.80%, and the average F1 score is 0.886.

For comparison purposes, the same diagnosis model of *K* = 2 was chosen to diagnose 200 sets of the fault instances that were not involved in the training. The diagnosis found that among 200 sets of the fault instances, 147 groups of the fault locations were correctly diagnosed, with a diagnostic accuracy rate of 73.5% and a diagnosis time of 12.16s.

Comparing Tables 3 and 4, it can be observed that Table 3 exhibits a better predictive performance of the model than Table 4. After the cross-validation, the average prediction accuracy, average hamming loss, average ranking loss, average coverage, average precision, average one-error, and average F1 score of this ventilation system resistance variant for the multiple fault locations diagnosis model were 72.95%, 2.62%, 6.04%, 256.33%, 88.53%, 3.80%, and 0.889, respectively. For the 200 groups of untrained fault diagnosis instances, the average diagnostic accuracy of the model was 75% and the average diagnostic time was 11.03s.

The total length of the roadways of the experimental metal production mine is about 30 kilometers. According to the average speed of one person walking 3 kilometers per hour without stopping, it will take at least 10 hours to check all the roadways of the mine. However, the fault diagnosis time of the resistance variant for the multiple fault locations diagnosis model for the ventilation system is less than one minute, and this shows that the model can quickly diagnose the locations of the faults when the ventilation system encounters the resistance variant multiple faults. Additionally, the model saves a lot of manpower and material resources. Most importantly, by quickly detecting the faults in the ventilation system, safety hazards can be eliminated promptly and the ventilation system can be restored to its normal production status, ensuring safe mine production.

## Conclusions

The ML-KNN-based diagnosis model of the mine ventilation system with the resistance variant for multiple fault locations has a remarkable prediction accuracy and precision. When the RVFs occur at multiple locations in the ventilation system, the locations of the roadways where the faults occur can be quickly and accurately determined. This allows dealing with the faults in time to restore the ventilation system to its normal state and prevent possible accidents to ensure a safe production in the mine.

The average prediction accuracy, average hamming loss, average ranking loss, average coverage, average precision, average one-error, and average F1 score of the ventilation system resistance variant for the multiple fault location diagnosis models after the cross-validation were 72.95%, 2.62%, 6.04%, 256.33%, 88.53%, 3.80%, and 0.889, respectively. This indicates that the model has a good prediction performance and can meet the field engineering requirements.

The current model diagnosed 200 groups of samples that did not take part in the training, where the average diagnosis accuracy was 75%, reflecting a good generalization ability. The average diagnosis time of the model is 11.03s, which is adapted to the emergency demand for mine ventilation system fault disposal.

Although utilizing the air volume as the observation information of the model can well diagnose the locations of the occurrence of the resistance variant multiple faults in the mine ventilation system, higher diagnostic accuracy can be achieved if the composite features are considered as the observation information. The air volume relies on the wind speed sensors, while the prediction performance of the diagnostic model using the air volume of all the branches of the ventilation network as input is slightly better than that using the air volume of residual branches as an input. It is unrealistic and uneconomical to deploy wind speed sensors in all the roadways of the ventilation system. Thus it is feasible to use the residual branches’ air volume of the ventilation network as the observation information for the fault locations diagnosis. However, not all the residual branches have suitable conditions to install the sensors. Therefore, the locations of the sensors must be optimized so that the sparse sensor readings of the ventilation system can be used to accurately diagnose the resistance variant for multiple faults in the ventilation system.

## Supporting information

S1 Table Topology data of the metal mine ventilation network. (PDF)

S1 File (PDF)
