# Supplementary material for: Intelligent diagnosis of resistance variant multiple fault locations of mine ventilation system based on ML-KNN
Source: PLoS One. 2022 Sep 30;17(9):e0275437. doi: 10.1371/journal.pone.0275437 (PMC9524657; doi:10.1371/journal.pone.0275437)
Supplement: S1 Table — (PDF) [file pone.0275437.s001.pdf]

SI Table. Topological data.

roadways				dampers		
id	s	t	r	id	eid	r
1	1	2	0.006441	1	87	0.285899
2	2	3	0.518656	2	88	1.281785
3	3	4	0.002141	3	64	4.506021
4	4	5	0.011815	4	212	0.517025
5	5	6	0.005236	5	60	10.06647
6	6	7	0.006001	6	55	14.73356
7	5	17	0.000605	7	9	3014.887
8	17	18	0.190744	8	213	4.294461
9	6	19	0.000485	9	40	0.28187
10	19	18	0.003847	10	42	3.177077
11	7	20	0.012792	11	43	1.322531
12	20	21	0.000065	12	217	976.1189
13	21	22	0.001631	13	216	1995.08
14	22	23	0.001309	14	215	0.411137
15	20	24	0.076134	15	160	15.7327
16	24	88	13.39528	16	221	1.075192
17	21	89	24.45939	17	7	1922.538
18	2	30	0.000843	18	23	0.248663
19	30	40	0.016797			
20	40	41	0.01533			
21	40	42	0.823957			
22	42	44	0.345965			
23	42	43	0.01438			
24	44	43	0.122076			
25	44	45	0.346831			
26	43	45	0.183654			
27	45	46	0.121427			
28	30	31	0.000475			
29	31	47	0.000061			
30	47	48	0.000519			
31	48	49	0.002231			
32	47	51	1.726473			
33	49	34	0.03325			
34	49	37	0.008914			
38	31	32	0.447867			
39	52	32	0.006481			
40	52	41	0.003676			
41	53	52	0.123248			
42	53	36	0.033815			
43	32	33	0.001554			
44	33	35	0.148492			
45	37	38	0.045421			
47	39	35	0.067493			
48	37	34	0.059425			
49	34	33	0.08115			
51	36	51	0.017717			
52	51	46	0.017717			

53	46	80	0.03189			
54	4	54	0.005489			
55	54	55	0.002676			
56	55	56	0.187337			
57	56	57	0.835811			
58	55	63	1.541622			
59	54	58	0.001365			
60	58	59	6.39E-05			
61	60	59	0.048975			
62	61	60	0.112265			
63	59	62	0.000623			
64	62	63	0.007724			
65	62	64	1.019636			
66	61	66	296.2805			
67	65	64	0.198048			
68	65	66	0.009071			
69	64	67	0.003267			
70	66	69	0.007227			
71	68	67	0.091947			
72	68	69	0.004643			
73	63	74	0.022867			
74	67	73	0.002852			
75	69	70	0.001331			
76	70	71	0.003298			
77	71	72	0.01476			
79	73	75	0.00319			
80	72	76	0.011195			
81	75	76	0.001184			
82	76	77	0.000206			
83	71	77	18.66382			
84	74	57	0.004314			
85	57	79	0.005608			
86	77	78	0.000542			
87	79	78	0.004134			
88	79	80	0.000753			
89	58	81	0.047799			
90	81	17	0.00415			
91	81	82	0.142033			
92	82	19	0.000582			
93	83	82	0.11326			
94	29	83	1.714154			
95	83	84	0.066043			
96	85	84	0.008108			
97	84	86	1.107972			
98	85	87	0.19167			
99	24	85	0.026253			
100	90	91	0.019007			
101	91	3	0.013624			
102	91	92	0.008632			
103	92	93	0.000061			

104	93	94	0.23705			
105	94	95	0.289314			
106	95	96	0.003992			
107	96	97	0.000516			
108	96	98	0.002314			
109	95	98	0.001167			
110	97	99	0.04165			
111	98	99	0.011364			
112	95	100	0.012642			
113	99	101	0.000292			
114	100	101	0.001615			
115	101	108	0.000725			
116	106	100	0.011488			
117	93	103	0.135383			
118	92	102	0.032968			
119	102	106	0.0217			
120	103	104	0.037017			
121	103	105	0.071143			
122	104	105	0.092115			
123	104	102	0.013562			
124	105	107	0.183418			
125	106	107	0.004605			
126	107	108	0.003686			
127	108	109	0.011252			
128	109	29	0.496395			
129	29	28	0.000756			
130	28	86	1.504731			
131	86	60	1.611348			
132	109	110	0.000371			
133	110	111	0.006073			
134	111	112	0.085128			
135	112	113	0.066983			
136	113	114	0.719807			
137	114	13	1.010841			
138	110	9	0.060191			
139	111	10	0.049123			
140	112	11	1.261563			
141	10	9	0.014065			
142	10	11	0.713964			
143	11	12	2.199233			
144	12	13	3.729924			
145	9	8	0.061391			
146	8	25	0.001329			
147	25	27	0.000265			
148	27	28	0.00158			
149	27	88	2.669876			
150	25	26	0.001584			
151	26	89	1.558805			
152	26	23	0.018541			
153	7	8	0.008192			

154	22	70	27.295			
155	23	72	1.850057			
156	89	68	1.669253			
157	88	65	3.097304			
158	87	61	9.357801			
160	116	117	0.004756			
161	117	118	235.8938			
162	118	119	49.81003			
163	118	120	21.7155			
164	121	119	0.019458			
165	121	120	0.010891			
166	120	126	0.040424			
167	122	121	0.001248			
168	122	123	0.000833			
169	124	122	0.017661			
170	136	124	0.016202			
171	124	125	0.005182			
172	125	123	0.004701			
173	125	126	0.001643			
174	119	499	0.012386			
175	123	127	0.031424			
176	126	128	0.096317			
177	127	128	0.014859			
178	128	500	0.057536			
179	129	130	0.014107			
180	117	131	170.0916			
181	129	131	0.023016			
182	131	130	0.020363			
183	116	132	0.006427			
184	132	133	0.143601			
185	133	135	0.00105			
186	133	134	0.002258			
187	135	136	0.004088			
188	141	136	0.022616			
189	134	135	0.005991			
190	134	137	0.205402			
191	132	138	0.005982			
192	138	141	0.258874			
193	138	139	0.161279			
194	139	140	0.252232			
195	140	141	0.084728			
196	139	142	0.03521			
197	142	140	0.037644			
198	142	137	10.27556			
199	137	130	0.061076			
200	130	92	0.016348			
201	113	14	5.453619			
202	13	14	2.712763			
203	14	701	0.076016			
204	80	143	0.163202			

205	78	144	0.043813			
206	143	144	0.028697			
207	144	15	0.003352			
208	143	700	0.01231			
209	15	145	0.000709			
211	145	146	0.003871			
212	18	75	0.66924			
213	41	56	0.096497			
214	12	114	4.704435			
215	147	148	0.006092			
216	148	149	0.04005			
217	148	150	0.036759			
218	148	53	0.09456			
219	115	151	0.218515			
220	151	116	0.101582			
221	151	94	0.005652			
222	38	150	0.000635			
223	150	39	0.007061			
224	35	149	0.009464			
225	149	36	0.165434			
498	499	127	0.015481			
499	499	500	0.537287			
500	500	129	0.006216			
700	700	145	0.01231			
702	701	15	0.023663			
701	700	701	0.023663			
