# Supplementary material for: Intelligent diagnosis of resistance variant multiple fault locations of mine ventilation system based on ML-KNN
Source: PLoS One. 2022 Sep 30;17(9):e0275437. doi: 10.1371/journal.pone.0275437 (PMC9524657; doi:10.1371/journal.pone.0275437)
Supplement: S1 File — (PDF) [file pone.0275437.s002.pdf]

TOPEDIT

TOPEDIT SCIENTIFIC EDITING

CERTIFICATE OF ENGLISH EDITING

This certificate confirms that the manuscript listed below was edited by one or more expert English editors with doctoral degree. The following issues were corrected: grammar, spelling, punctuation, sentence structure, and phrasing. Journal editors can contact us at info@topeditsci.com if you have any concerns.

Manuscript title

Intelligent Diagnosis of Resistance Variant M ultiple Fault Locations of Mine Ventilation Sy stem Based on ML-KNN

Date issued

05/13/2022

Certificate number

CN 8071-22-0513-02

TopEdit specializes in comprehensive preparation of scientific manuscripts that are targeted for publication in English language journals. We provide editing, technical translation, formatting, and illustration services for technical manuscripts, grant proposals, conference presentations and posters, and professional school application essays and other scientific materials. All orders are edited by one or more editors who hold advanced Ph.D. degrees and are either natively proficient in English or native speakers.

https://www.topeditsci.com
